# Supplementary material for: The Small RNA Universe of Capitella teleta
Source: Front Mol Biosci. 2022 Feb 25;9:802814. doi: 10.3389/fmolb.2022.802814 (PMC8915122; doi:10.3389/fmolb.2022.802814)
Supplement: Supplementary file 1 [file DataSheet1.ZIP › Supplement/known/CAPTEscaffold_591_25240.pdf]

Provisional ID	:	CAPTEscaffold_591_25240
Score total	:	853.4
Score for star read(s)	:	3.9
Score for read counts	:	843.4
Score for mfe	:	1.5
Score for randfold	:	1.6
Score for cons. seed	:	3
Total read count	:	1666
Mature read count	:	1647
Loop read count	:	0
Star read count	:	19

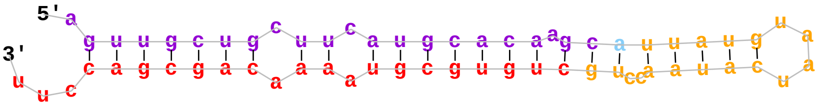

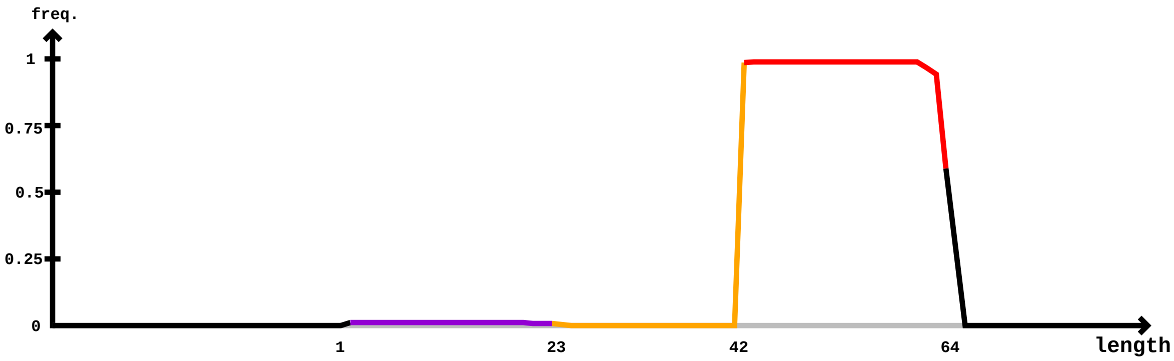

Star Mature

```
5'- ggcaugagaguguccucggcugucacugcaguugcugcuucaugcacaagcauuauguaaucauaaccugcugugcguaaaacagcgaccuuugugggagguagaaaucaua  -3'  obs
    ggcaugagaguguccucggcugucacugcaguugcugcuucaugcacaagcauuauguaaucauaaccugcugugcguaaaacagcgaccuuugugggagguagaaaucaua       exp
    ggcaugagaguguccucggcugucacugcaguugcugcuucaugcacaagcauuauguaaucauaaccugcugugcguaaaacagcgaccuuugugggagguagaaaucaua       known
    (((((....)))))((.(.((.((((....(((((((.((.(((((((.((((((((....)))))..)))))))))).)).)))))))....)))).)).)))........  reads  mm  sample
    .............................aguugcugcuucaugcaca................................................................  6      0   seq
    .............................aguugcugcuucaugcacaagc.............................................................  9      0   seq
    .............................aguugcugcuucaugcacaagca............................................................  4      0   seq
    ......................................................................cugugcguaaaacagcgaU.......................  1      1   seq
    ......................................................................cugugcguaaaacagcgac.......................  36     0   seq
    ......................................................................cugugcguaaaacagcgacA......................  1      1   seq
    ......................................................................cugugcguaaaacagcgacc......................  39     0   seq
    ......................................................................cugugcguaaaacGgcgaccu.....................  1      1   seq
    ......................................................................cugugcguaaGacagcgaccu.....................  1      1   seq
    ......................................................................cugugcguaaaaUagcgaccu.....................  1      1   seq
    ......................................................................cugugcguaaaacagcAaccu.....................  1      1   seq
    ......................................................................cugugcguaaaacagcgaccG.....................  1      1   seq
    ......................................................................cugugcguaaaacagcgaccu.....................  581    0   seq
    ......................................................................cAgugcguaaaacagcgaccu.....................  1      1   seq
    ......................................................................cugugcguaaaacagcgacAuu....................  2      1   seq
    ......................................................................cugugcguaaaacagcgaccuA....................  2      1   seq
    ......................................................................cugugcguaaaacagcgaccuu....................  931    0   seq
    ......................................................................cugugcguaaaacagcgaccCu....................  1      1   seq
    ......................................................................cugugcguaaaacagcgaccuG....................  2      1   seq
    ......................................................................cugugcgCaaaacagcgaccuu....................  1      1   seq
    ......................................................................cugugcguaaaUcagcgaccuu....................  1      1   seq
    ......................................................................cugugcguaaaacGgcgaccuu....................  1      1   seq
    ......................................................................cuguUcguaaaacagcgaccuu....................  1      1   seq
    ......................................................................cugugcguaaaacagcgaccuuu...................  34     0   seq
    ......................................................................cugugcguaaaacagcgaccuuA...................  3      1   seq
    .......................................................................ugugcguaaaacagcgaccu.....................  2      0   seq
    .......................................................................ugugcguaaaacagcgaccuu....................  1      0   seq
    .......................................................................ugugcguaaaacagcgaccAu....................  1      1   seq
```
